# Early and Late Pathogenic Events of Newborn Mice Encephalitis Experimentally Induced by *Itacaiunas* and *Curionópolis* Bracorhabdoviruses Infection

**DOI:** 10.1371/journal.pone.0001733

**Published:** 2008-03-05

**Authors:** José Antonio Picanço Diniz, Zaire Alves dos Santos, Marcio Augusto Galvão Braga, Ádila Liliane Barros Dias, Daisy Elaine Andrade da Silva, Daniele Barbosa de Almeida Medeiros, Vera Lucia Reis de Souza Barros, Jannifer Oliveira Chiang, Kendra Eyllen de Freitas Zoghbi, Juarez Antônio Simões Quaresma, Christina Maeda Takiya, Vivaldo Moura Neto, Wanderley de Souza, Pedro Fernando da Costa Vasconcelos, Cristovam Wanderley Picanço Diniz

**Affiliations:** 1 Instituto Evandro Chagas, Belém, Brazil; 2 Universidade Federal do Pará, Belém, Brazil; 3 Universidade Federal do Rio de Janeiro, Rio de Janeiro, Brazil; Cambridge University, United Kingdom

## Abstract

In previous reports we proposed a new genus for Rhabdoviridae and described neurotropic preference and gross neuropathology in newborn albino Swiss mice after Curionopolis and Itacaiunas infections. In the present report a time-course study of experimental encephalitis induced by Itacaiunas and Curionopolis virus was conducted both in vivo and in vitro to investigate cellular targets and the sequence of neuroinvasion. We also investigate, after intranasal inoculation, clinical signs, histopathology and apoptosis in correlation with viral immunolabeling at different time points. Curionopolis and Itacaiunas viral antigens were first detected in the parenchyma of olfactory pathways at 2 and 3 days post-inoculation (dpi) and the first clinical signs were observed at 4 and 8 dpi, respectively. After Curionopolis infection, the mortality rate was 100% between 5 and 6 dpi, and 35% between 8 and 15 dpi after Itacaiunas infection. We identified CNS mice cell types both in vivo and in vitro and the temporal sequence of neuroanatomical olfactory areas infected by Itacaiunas and Curionopolis virus. Distinct virulences were reflected in the neuropathological changes including TUNEL immunolabeling and cytopathic effects, more intense and precocious after intracerebral or in vitro inoculations of Curionopolis than after Itacaiunas virus. In vitro studies revealed neuronal but not astrocyte or microglial cytopathic effects at 2 dpi, with monolayer destruction occurring at 5 and 7 dpi with Curionopolis and Itacaiunas virus, respectively. Ultrastructural changes included virus budding associated with interstitial and perivascular edema, endothelial hypertrophy, a reduced and/or collapsed small vessel luminal area, thickening of the capillary basement membrane, and presence of phagocytosed apoptotic bodies. Glial cells with viral budding similar to oligodendrocytes were infected with Itacaiunas virus but not with Curionopolis virus. Thus, Curionopolis and Itacaiunas viruses share many pathological and clinical features present in other rhabdoviruses but distinct virulence and glial targets in newborn albino Swiss mice brain.

## Introduction

Many efforts have been dedicated to understand the neuropathogenesis of experimental viral encephalitis [1], [2], [3], [4], [5]. However, few studies have investigated the experimental neuropathology of arboviruses [6], [7], [8], [9], [10], and virtually no studies have addressed the neuropathology of emerging Amazonian rhabdoviruses [11], [12]. The family Rhabdoviridae is characterized by its unique morphology of virus particles and consists of six genera: *Vesiculovirus*, *Lyssavirus*, *Ephemerovirus*, *Novirhabdovirus*, *Cytorhabdovirus*, *and Nucleorhabdovirus* [13]. More recently, a new genus, called *Bracorhabdovirus*, has been proposed for that family based on the nucleotide sequencing, serological reactions and ultrastructural morphology of two virus species, Itacaiunas and Curionopolis viruses [14]. These species were isolated from *Culicoides* midges in the municipality of Parauapebas, State of Pará, Brazil, in 1984 and 1985, respectively. A previous report described the neurotropic preference and neuropathological outcomes of these rhabdoviruses in newborn mice with severe experimental encephalitis [11].

The pathogenesis of viral invasion of the central nervous system (CNS) involves many distinct steps, including replication at the primary site of infection, entry into and spread within the CNS, replication in neural tissue, host immune response, and tissue injury [15], [2], [4]. Many species of the family Rhabdoviridae have been used successfully to induce experimental encephalitis in mice [11], [16], [17], [18]. The genus *Vesiculovirus* has been used as a model *in vitro* and *in vivo* studies investigating viral adaptive and host immune responses [19]. When *Vesicular stomatitis Indiana virus* (VSIV) was inoculated intranasally into 5–7-week-old male BALB/c mice, olfactory receptor neurons were the first cells to be infected [10], [20], followed by neurons of the olfactory bulb and, finally, acute infection of other brain areas [21], [8], [22]. Complete clearance of viral antigens from the brain parenchyma was observed in surviving mice within 12 days post-inoculation (dpi), without long-term damage to the brain [21], [8].

At present, little is known about the interactions of Itacaiunas and Curionopolis viruses with neuronal or glial cells possibly affected during the temporal course of encephalitis. In previous reports we proposed a new genus for Rhabdoviridae [14] and described neurotropic preference and gross neuropathology in newborn albino Swiss mice after Curionopolis and Itacaiunas infections [11]. In the present report a time-course study of experimental encephalitis induced by Itacaiunas and Curionopolis virus was conducted both in vivo and in vitro to investigate cellular targets and the sequence of neuroinvasion. We also investigate, after intranasal inoculation, clinical signs, histopathology and apoptosis in correlation with viral immunolabeling at different time points.

Thus, the main objective of the present study was to investigate details of the pathogenesis of these neurotropic viruses in a time-course experiment after *in vivo* and *in vitro* inoculation, to answer the following questions in the newborn albino Swiss mice:

1. What are the cell targets and the sequence of neuroinvasion?
2. Does a correlation exist between the severity of clinical signs and intensity of viral antigen immunolabeling in the parenchyma?
3. Is there any specific difference in the ultrastructural damage induced by each virus that may distinguish possible neuropathological and clinical outcomes?

## Methods

### 1. Animals, Viral Species and Infection

The suckling Swiss mice used in our study were obtained from the animal care facility at Instituto Evandro Chagas (IEC) and maintained in standard mouse cages. All animal procedures were in accordance with the National Institutes of Health Guide for the Care and Use of Laboratory Animals (2nd Edition, 2002) and Brazilian laws, and were performed in biosafety level 2 facilities. A different subset of adult animals (n = ?) were also infected by intranasal inoculation but did not present detectable clinical signs despite the presence of viral antigenic immunolabeling in the brain. The study was approved by the Ethics Committee of IEC. The selected viral strains were characterized as members of the family Rhabdoviridae and of the proposed new genus, *Bracorhabdovirus*, based on their biological, antigenic, serological, and genetic characteristics according to protocols described elsewhere [11], [22].

Virus-containing brain homogenates were obtained as follows: 0.02 ml of each viral suspension was first inoculated intracerebrally into newborn mice and the animals were observed daily. When presenting clinical signs, the animals were sacrificed and immediately stored at −70°C. Later, brain tissue (0.2 g/animal) was macerated and mixed with 1.8 ml phosphate-buffered saline (PBS) containing 100 U/ml penicillin and 100 µg/ml streptomycin. The suspension was cleared by centrifugation at 10,000 *g* for 15 min at 4°C. Virus titration was carried out by intracerebral inoculation of newborn mice with 0.02 ml of serial 10-fold dilutions of the viral suspensions in PBS, and LD_50_ values were calculated [24].

### 2. In Vivo Assays

#### 2.1 Experimental groups and inoculation

*Infected groups*: Supernatants of the viral suspensions were inoculated intranasally (5 µl of each virus suspension into the two nostrils of newborn mice using a 10-µl micropipette). After inoculation, the animals were observed daily and sacrificed and perfused at the survival times chosen according to virus species (Curionopolis: 1, 2, 4 and 6 dpi; Itacaiunas: 1, 2, 4, 6, 8, 12, 15, 23 and 30 dpi). A total of 156 (2–3 days old) animals were inoculated intranasally with 0.005 ml (1∶2, v/v) of one of the two viruses (Itacaiunas virus: n = 108; Curionopolis virus: n = 48).

*Intracerebral inoculation:* Newborn mice (2–3 days old) were also inoculated intracerebrally with 0.02 ml of a viral suspension containing homogenized infected mouse brains diluted 1∶10 (v/v) in PBS containing 0.75% bovine serum albumin and antibiotics. Newborn mice infected with Curionopolis virus became sick and died within 3–4 dpi, while Itacaiunas virus killed newborn mice at 4–5 dpi. The Itacaiunas and Curionopolis virus titers were 4.7 log10 and 5.6 log10, respectively.

*Control groups:* Another set of animals (n = 78) were used as uninfected controls and observed under the same conditions as infected animals.

#### 2.2 Perfusion and microtomy

Animals were anesthetized by hypothermia (up to 4 days of age) or with Avertin (6 days of age or older) and perfused intracardially through the left ventricle with 0.9% saline and 4% formaldehyde in 0.1 M PBS, pH 7.2. After craniotomy, the brains were post-fixed in the primary fixative solution for 48 h and cut into 150-µm thick sections with a Vibratome (Micron International GmbH, Walldorf, Germany). Alternatively, fixed tissue was processed for gross histopathology after paraffin embedding. Blocks were cut into 10-µm thick sections with a microtome (Leica Microsystems, Inc., Bannockburn, IL, USA), mounted on electrostatic slides and stained with hematoxylin-eosin.

#### 2.3 Immunocytochemistry

*Viral antigen detection:* In order to assess the distribution of Curionopolis and Itacaiunas viral antigens in newborn mouse brain at different time points, immunohistochemistry was performed on all infected brains. Specific antibodies against each virus species were produced at IEC according to protocols published elsewhere [14], [23]. Free-floating sections were rinsed once in 0.05 M PBS for 5 min. The sections were incubated under constant shaking in 1% hydrogen peroxide solution in methanol for 20 min, rinsed in a 0.05% Tris-buffer saline (TBS)/Tween (Sigma, St. Louis, MO, USA) solution for 5 min (4 times), and incubated with 10% normal horse serum in PBS for 30 min. The sections were then Ig blocked according to the instructions of the Mouse-on-Mouse Immunodetection kit (M.O.M. kit, Vector Laboratories, Burlingame, CA, USA) and incubated with the primary antibody diluted in protein-concentrated solution (M.O.M. kit) overnight. Next, the sections were washed 4 times in PBS/Tween solution for 5 min and incubated for 1 h with the biotinylated horse anti-mouse secondary antibody (M.O.M. kit) diluted 1∶100 in PBS. As a negative control, normal horse serum rather than the primary antibody was added to some slides for each antibody used for each virus. The slides were rinsed again for 5 min (4 times) and incubated with the avidin/biotin/peroxidase complex (M.O.M. kit) according to manufacturer instructions. The sections were then rinsed 4 times (5 min each rinse) and the reaction was developed with 3, 3′-diaminobenzidine tetrahydrochloride dihydrate (DAB buffer tablets, Merck, Darmstadt, Germany) according to manufacturer instructions. Color was detected and sections were rinsed 4 times (5 min each) in 0.1 M PBS, embedded in n-propyl gallate, and coverslipped.

*Apoptosis detection:* In order to investigate cell death by apoptosis induced by Curionopolis and Itacaiunas rhabdoviruses in infected newborn mice at different time points, immunohistochemistry for caspase-3 and terminal deoxynucleotidyl transferase-mediated dUTP nick end-labeling (TUNEL) assay for specific apoptosis markers were performed [25], [26]. The primary antibody for caspase-3 (Promega, Madison, WI, USA) was diluted 1:250 in PBS. Alternatively, sections were submitted to the TUNEL assay at various time points to determine DNA fragmentation according to manufacturer instructions (In Situ Cell Death Detection kit, POD, Roche Diagnostics, Mannheim, Germany). In brief, antigen retrieval was performed by incubating the sections in 0.1 M citrate buffer, pH 6.0, for 15 min. Endogenous peroxidase was blocked by incubation with 3% H_2_O_2_ in 100% methanol for 5 min. Nonspecific epitopes were blocked with a mixture of 0.1 M Tris-buffer, pH 7.5, 3% bovine serum albumin and 20% normal bovine serum for 1 h. More permeable tissue was obtained by incubating the sections in 0.2% Triton X-100 in 0.1 M sodium citrate buffer, pH 6.0, for 30 min. The sections were then incubated in the labeling solution for 2 h and washed 3 times in 0.1 M PBS for 5 min each according to manufacturer instructions. Next, the sections were incubated with the converter reagent (POD kit) for 30 min and washed in 0.1 M PBS (3 times, 5 min each) and the reaction was developed with DAB/peroxidase.

The remaining immunohistochemical steps for apoptosis detection were similar to those described above. Some sections were also counterstained with cresyl violet.

### 3. In Vitro Assays

Immunofluorescence assays were performed on all types of cell cultures used in this study according to a previously described protocol [27]. Analysis of cross-reactivity in the immunofluorescence assay using Curionopolis and Itacaiunas antigens and hyperimmune mouse serum showed that these viruses only reacted with their respective antiserum [14].

#### 3.1 Preparation of primary mouse neuron and glial cultures

Primary cultures of neurons were prepared from mouse embryos using a previously published protocol, with slight modifications [28]. In brief, a pregnant (16–18 days of gestation) mouse was killed under halothane anesthesia. The uterus with embryos was immediately extracted under sterile conditions and placed in a 100-mm Petri dish containing cold, sterile Hanks' balanced salt solution (HBSS; Gibco BRL, Grand Island, NY, USA), supplemented with 1.0 mM sodium pyruvate (Sigma, St. Louis, MO, USA) and 10 mM HEPES (Gibco BRL, Grand Island, NY, USA), pH 7.4. The brains were removed from the embryos, transferred to a new dish containing cold HBSS, and meninges and blood vessels were carefully removed. Individual cells were isolated by trituration in cold HBSS using a fire-polished Pasteur pipette. After non-dispersed tissue was allowed to settle for 35 min, the supernatant was centrifuged for 5 min at 350 *g*. The cell pellet was resuspended in Neurobasal medium (Gibco BRL, Grand Island, NY, USA) supplemented with 1 mM Glutamax (Gibco BRL, Grand Island, NY, USA), 25 µM glutamate (Sigma), and B27 (2 ml of a 50× concentrate; Gibco BRL, Grand Island, NY, USA). The cells were either plated on glass coverslips in multi-well dishes (24 wells) (TPP, Switzerland) or transferred to 25-cm^2^ plastic flasks (TPP) at a concentration of 2×10^5 ^cells/ml in Neurobasal medium. The cultures were usually inoculated 3 to 4 days after plating of the cells. Mixed primary glial cultures were prepared as described previously [29], with slight modifications. Briefly, the brains of anesthetized 2-day-old mouse pups were removed and disrupted by mechanical trituration in sterile PBS supplemented with 0.6% D-glucose (Sigma, St. Louis, MO, USA). After non-dissociated tissue was allowed to settle for 3–5 min, the cell supernatant was centrifuged for 5 min at 350 *g*. The pellet was resuspended in Dulbecco's modified Eagles medium/nutrient mixture F-12 (Gibco BRL, Grand Island, NY, USA) supplemented with 10% fetal bovine serum (Gibco BRL, Grand Island, NY, USA), 2 mM glutamine (Sigma, St. Louis, MO, USA), 0.6% D-glucose (Gibco BRL, Grand Island, NY, USA), 3 mM sodium bicarbonate (Gibco BRL, Grand Island, NY, USA), and 0.5 mg/ml penicillin-streptomycin (Gibco BRL, Grand Island, NY, USA), henceforth referred to as “glial medium”. The dissociated cells were transferred to 25- or 75-cm^2^ plastic culture flasks at a concentration of 2×10^5^ cells/ml in glial medium. These cultures usually reached confluence within 10–14 days.

#### 3.2 Primary astrocyte cultures

The procedures for preparation of primary mouse astrocyte cultures were adapted from a previously described protocol [29]. Briefly, 10–14 days after plating of glial cells, 75-cm^2^ flasks containing confluent cells were rinsed in glial medium and mechanically shaken at 250 rpm for 18 h in an orbital shaker incubator (combi-SV 12 hybridization incubator, Finemould Precision Ind. Co.; Yang-Chun, Seoul, Korea) at 37°C. After shaking, the supernatant mainly consisted of oligodendrocytes and microglia detached during shaking. The glial medium was replaced onto a predominantly astrocyte monolayer. After 3 days, the flask-adherent astrocytes were trypsinized, collected, and either plated onto glass coverslips in multi-well dishes (24 wells; TPP) or transferred to 25-cm^2^ flasks at a concentration of 2×10^5^ cells/ml in glial medium.

#### 3.3 Primary microglial cultures

Microglial cultures were obtained following a protocol described elsewhere [30], with minor modifications. Briefly, isolated bright cells located over the culture monolayer of culture flasks were obtained after rinsing and manual shaking, followed by centrifugation of the supernatant at 350 *g* for 5 min. After resuspension, microglial cells were plated and maintained in glial medium as described previously.

Virus inoculation of cell cultures was performed using a viral suspension containing homogenized infected mouse brains at a final concentration of 1∶100 (v/v) in culture medium.

All glass coverslips and culture flasks were treated previously with a solution of 6.25 µg/ml cold poly-L-ornithine (0.16 ml/cm^2^ surface area) overnight. All cultures were incubated at 37°C in a humidified 5% CO_2_ atmosphere and cells were observed daily with an Axiovert S100 (Zeiss) microscope.

### 4. Transmission electron microscopy analysis

Primary cultures of neurons and mouse brains infected or not with Curionopolis or Itacaiunas virus were processed to obtain ultrathin sections and analyzed with a Zeiss EM 900 transmission electron microscope as described elsewhere [14]. In brief, immediately after removal of the medium, cell monolayers (infected and noninfected) were fixed for 2 h in a mixture of 4% formaldehyde and 2.5% glutaraldehyde in 0.1 M sodium cacodylate buffer, pH 7.2, containing 5 mM CaCl_2_ at room temperature. After primary fixation, the monolayers were washed in 0.1 M cacodylate buffer. The cells were scraped off the plastic, pelleted by centrifugation (5500 *g* for 5 min) in buffer, and post-fixed in a solution containing 1% osmium tetroxide, 0.8% potassium ferrocyanide and 5 mM CaCl_2_ [31] at room temperature in the same buffer. Cells were stained *en bloc* with 2% uranyl acetate in 25% acetone, dehydrated in graded acetone concentrations, and embedded in EMbed-812 (Electron Microscopy Sciences, Fort Washington, PA, USA). Samples obtained from mouse brains after perfusion and craniotomy were cut into small fragments and immersed for 2 h in a fixative solution containing 2.5% glutaraldehyde in 0.1 M PBS, pH 7.2, at room temperature. All other technical steps following fixation were similar to those described for *in vitro* samples. Ultrathin sections were obtained with a Reichert/Leica Ultracut S ultramicrotome (Leica Microsystems, Bannockburn, IL, USA) and stained with aqueous uranyl acetate and lead citrate [32] before examination.

### 5. Photomicrographs and Image Processing

Photomicrographs were obtained with a digital camera coupled to a Zeiss Microscope (AxioCam HRc, Zeiss, Jena, Germany). The brightness and contrast of the images were adjusted with the Adobe Photoshop 7.0.1 cs2 software (San Jose, CA, USA). Bright-field, interferential contrast and fluorescence photomicrographs were obtained with a light microscope (Zeiss-Axiophot), whereas phase-contrast images were taken with an inverted microscope (Zeiss-Axiovert S100).

## Results

After Itacaiunas and Curionopolis virus infections, the cellular targets both in vivo and in vitro and the sequence of neuroinvasion are described in association with clinical symptoms, in a time course study in newborn mice. These viruses did not induce encephalitis in mature albino Swiss mice but exhibit antigenic immunolabeling in olfactory pathways in non-symptomatic subjects (not illustrated).

### *In Vitro* Assays

**Figure 1:**
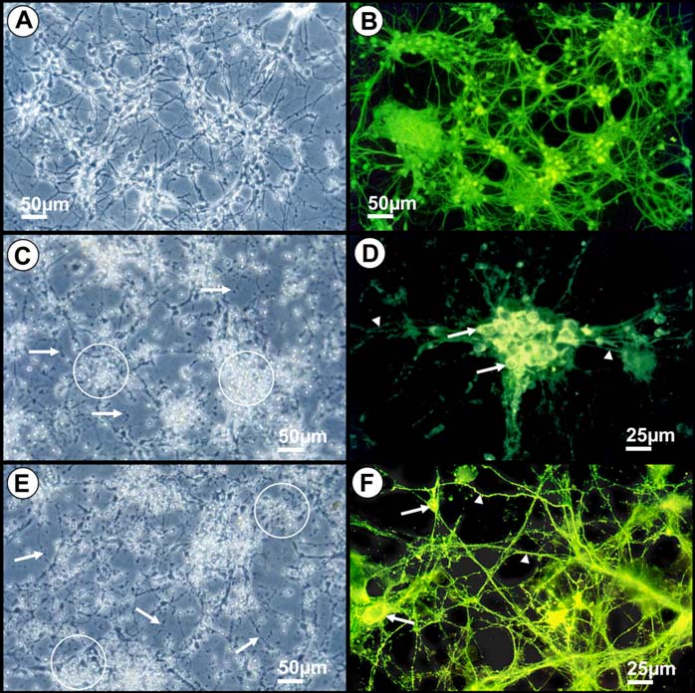
Phase-contrast (A, C, E) and fluorescence photomicrographs (B, D, F) of primary neuronal cultures to illustrate normal noninfected cell morphology (A, B) and cytopathic effects 3 days after inoculation of Curionopolis virus (C, D) and 4 days after inoculation of Itacaiunas virus (E, F). Arrows point to neurite fragmentation and circles indicate refringent points probably corresponding to apoptotic nuclei (A, C). Arrows and arrowheads indicate infected soma and dendrites, respectively (D, F). Neurons were stained by indirect immunofluorescence for anti-neurofilament antibodies. Secondary antibodies were conjugated with fluorescein isothiocyanate.

Figure 1 illustrates the indirect immunofluorescent staining for neurofilaments of neurons in primary cell cultures. The typical morphology of neurons can be compared with the altered features of infected cultures. Infected and normal neurons were detected *in vitro* by indirect immunofluorescence and cytopathic effects of Curionopolis and Itacaiunas viruses were observed after 3 and 4 dpi, respectively. Cytopathic effects were evident and consisted of neurite fragmentation and refringent points probably indicating apoptotic nuclei. At 5 and 7 dpi, the Curionopolis and Itacaiunas viruses had completely destroyed the neuronal monolayer, respectively. Primary astrocyte and microglial cell cultures were examined by indirect immunocytochemistry for glial fibrillary acid protein and fluorescein B4 isolectin, respectively, and no cytopathic effect was observed (data not shown).

**Figure 2:**
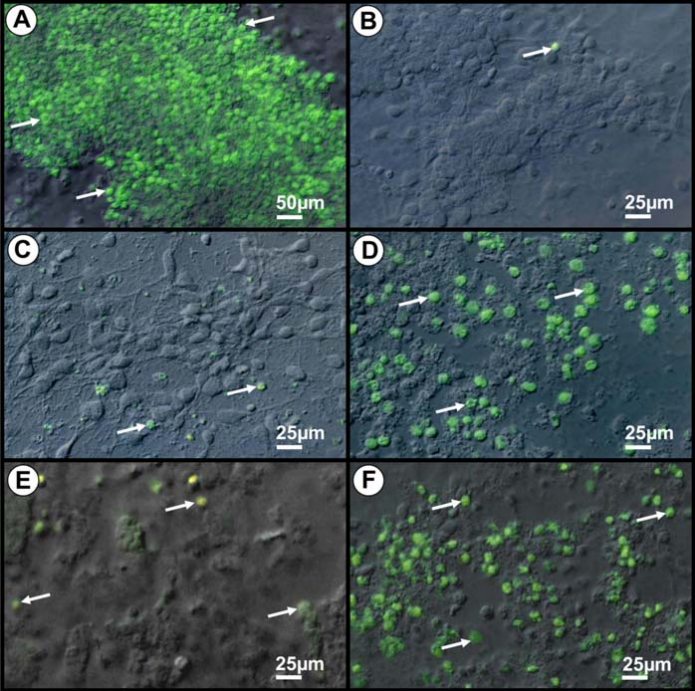
Combined interferential contrast and fluorescent photomicrographs of positive and negative controls (A, B) and Curionopolis- (C, D) or Itacaiunas (E,F)-infected neurons after TUNEL immunolabeling. Apoptotic nuclei (arrows) are observed in control cultures exposed to UV light (A) and after Curionopolis (D) and Itacaiunas (F) virus inoculation at 4 and 5 days post-inoculation, respectively. Negative control cultures (B) and infected cultures at 1 day post-inoculation (C, E) present few stained nuclei.

Figure 2 illustrates the TUNEL immunocytochemical reactions for UV exposed noninfected and infected neuronal cultures. Infected cultures corresponded to 4 and 5 dpi with Curionopolis and Itacaiunas virus, respectively. As expected, positive control cultures exhibited a massive amount of TUNEL-positive cells after UV exposure when compared to non-exposed control cultures. Inoculation with the two viral species induced neuronal TUNEL immunolabeling, but at an earlier stage for Curionopolis virus compared to Itacaiunas virus infection.

**Figure 3:**
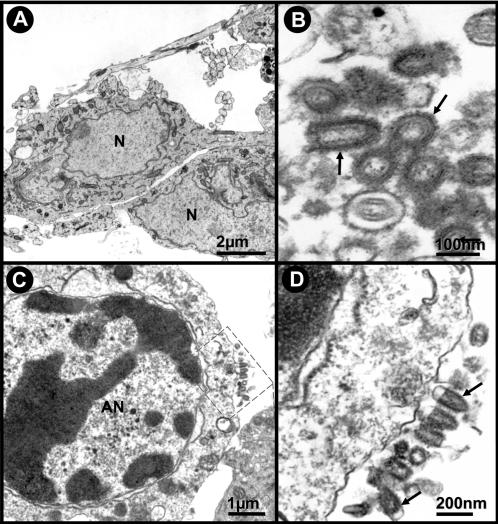
Transmission electron photomicrographs of ultrathin sections obtained from primary neuronal cultures. Control cultures of normal cells (A) and after Itacaiunas (B) and Curionopolis (C, D) infection at 4 and 5 days post-inoculation, respectively. Apoptotic cells (C) and virus budding (rectangle and arrows) (C, D) after Curionopolis infection. Itacaiunas virus particles (arrows) in culture at 5 days post-inoculation (B). N: nucleus; AN: apoptotic nucleus.

Figure 3 illustrates the ultrastructural neuronal and viral morphology of cell cultures inoculated with Curionopolis and Itacaiunas virus. The neuronal apoptotic morphology showing viral membrane budding of Curionopolis (4 dpi) and Itacaiunas (5 dpi) virus particles into the extracellular space is illustrated. The typical bullet morphology was found in both species as reported previously [14]. The presence of viral membrane budding and apoptotic cell features suggests that viral replication and the mechanisms of death of infected cells may be similar to those described for other rhabdoviruses [33], [34].

### *In Vivo* Assays

#### *Early and Late Neuropathological Features, Clinical Signs, and Death*

*Intracerebral inoculation*

*Light microscopy level*

**Figure 4:**
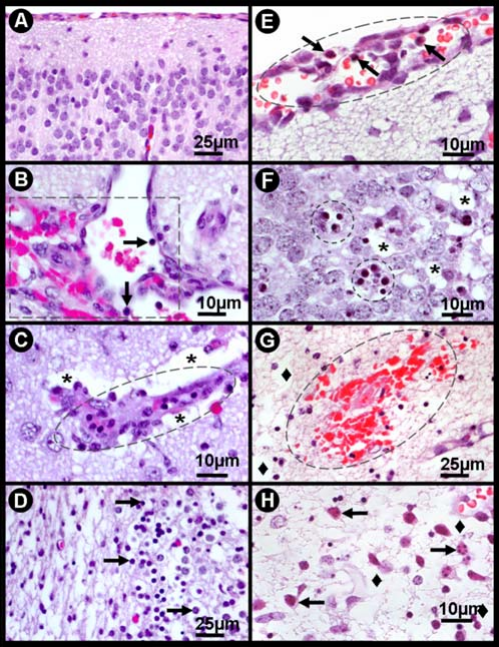
Photomicrographs of hematoxylin-eosin-stained sections from control (A) and infected brain sections at 96 h post-inoculation with Itacaiunas virus (B–D) and 60 h post-inoculation with Curionopolis virus (E–H). Multiple foci of congestion with sparse distribution of leukocytes characterized by the margination phenomenon (leukocytes becoming flat and the plasma membrane sticking to the capillary endothelium) (B); hypertrophic endotheliocytes (ellipse) and perivascular edema (stars) (C). Apoptotic like-cells with nuclear pyknosis and cytoplasmic condensation were detected among tissue vacuolation (arrows) (D). Meningeal congestion and edema with lymphomononuclear cells (arrows) (E). Groups of pyknotic cells presenting cytoplasmic condensation (circles), mixed with normal cells and vacuolated parenchyma (stars) (F). Recent hemorrhagic points (presence of red blood cells) presenting parenchymatous edema (lozenge), mainly in the areas of worst cell damage (84 h post-inoculation) (G). Nuclear fragmentation or karyorrhexis (arrows) and edema (lozenge) (96 h post-inoculation) (H).

Figure 4 shows the neuropathological features after intracerebral infection with Itacaiunas (A–D) and Curionopolis (E–H) virus. Meningeal congestion, parenchymatous edema with multiple foci of congestion and altered leukocytes characterized by a flat morphology and plasma membrane adhering to the capillary endothelium (margination phenomenon) were observed 96 h after infection with Itacaiunas virus. Hypertrophic endotheliocytes and perivascular edema were sometimes associated with intense vascular congestion and hemorrhagic foci were associated with leukocyte infiltration. Apoptotic like-cells presenting nuclear pyknosis and cytoplasmic condensation were also detected among tissue vacuolation. Neuronal pyknosis, cellular rarefaction, vacuolation and nuclear fragmentation were frequent pathological features in damaged areas 60 h after inoculation of Curionopolis virus. Meningeal congestion accompanied by lymphomononuclear cells, perivascular edema and endothelial hypertrophy was also noted. Hemorrhagic foci associated with parenchymatous edema were observed 84 h post-inoculation, mainly in the areas of worst cell damage (data not shown). Nuclear fragmentation or karyorrhexis and edema predominated at 96 h post-inoculation.

At 96 h post-inoculation, intense pyknosis, cellular rarefaction, apoptosis and an inflammatory tissue reaction were observed in both Curionopolis and Itacaiunas infections but were much more intense in brains infected with Curionopolis virus. Intense TUNEL immunolabeling was detected for both Curionopolis and Itacaiunas virus 72 h post-inoculation, with a much larger number of labeled cells being observed in brain sections inoculated with Curionopolis virus (data not shown).

*Electron microscopy level*

**Figure 5:**
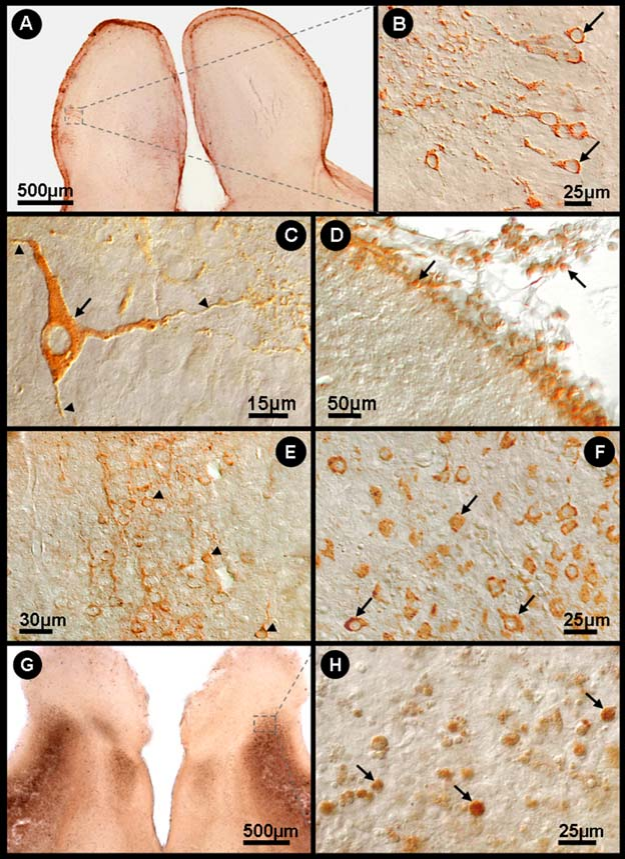
Transmission electron photomicrographs of ultrathin sections obtained from control (A) and mouse brain infected intracerebrally with Curionopolis for 36 (B,C), 60 (D) and 96 h (E), and with Itacaiunas for 24 (F), 60 (G), 72 (H), 96 (I) and 108 h (J). Normal tissue with intact neuronal soma and appendages (A); viral particles (arrow), interstitial edema (stars) and cellular rarefaction (lozenge) are seen 36 h post-inoculation (p.i.) (B, C); necrotic cells were observed at 60 h p.i. (D); intense perivascular edema (stars), hyperplastic endotheliocytes and reduced vessel luminal area (E); well-preserved brain parenchyma and vessels at 24 h p.i. (F); viral particles, endotheliocyte hyperplasia, and mild interstitial edema (stars) at 60 h p.i. (G); membrane viral budding in rich polyribosomes oligodendrocyte-like cell at 72 h p.i. (H); brain parenchyma at 96 h p.i. presenting a large number of viral particles (I); apoptotic features were more marked at 108 h p.i. (J). AC = apoptotic cell, M = mitochondria, OL = oligodendrocyte, EC = endothelial cells, VL = vascular lumen, N = cell nucleus, NC = necrotic cells.

Figure 5 shows electron microscopic images of normal tissue (A) and neuropathological features after intracerebral inoculation of Curionopolis (B–E) and Itacaiunas (F–J) virus into the cerebral cortex at different time points. Virus budding from the neuronal cell membrane, interstitial edema and cellular rarefaction were observed 36 h after Curionopolis inoculation. Necrotic cells were first detected at 60 h post-inoculation. After 96 h, intense perivascular edema and endothelial hyperplasia accompanied by a marked reduction in the luminal area of blood vessels were noted (data not shown). There were no glial cells infected with Curionopolis virus. Electron microscopy analysis of the cerebral cortex (F–J) revealed no change 24 h after intracerebral infection with Itacaiunas virus. Viral replication at an early stage was detected 60 h post-inoculation. After 72 h, glial cells containing abundant cytoplasmic polyribosomes, similar to oligodendrocytes, presented dilatations of the rough endoplasmic reticulum and viral budding. At 84 h post-inoculation, Itacaiunas virus-infected tissue was characterized by interstitial edema and cellular rarefaction around the capillary bed, pericytes with phagosomes, mitochondrial membrane rupture, dilatation of the rough endoplasmic reticulum of endothelial cells, and presence of phagosomes (data not shown). At 96 h post-inoculation, large numbers of viral particles were detected in brain parenchyma, and intense cellular rarefaction and interstitial edema were observed in large areas. Apoptotic neurons were the predominant finding at 108 h post-inoculation.

*Intranasal inoculation*

**Figure 6:**
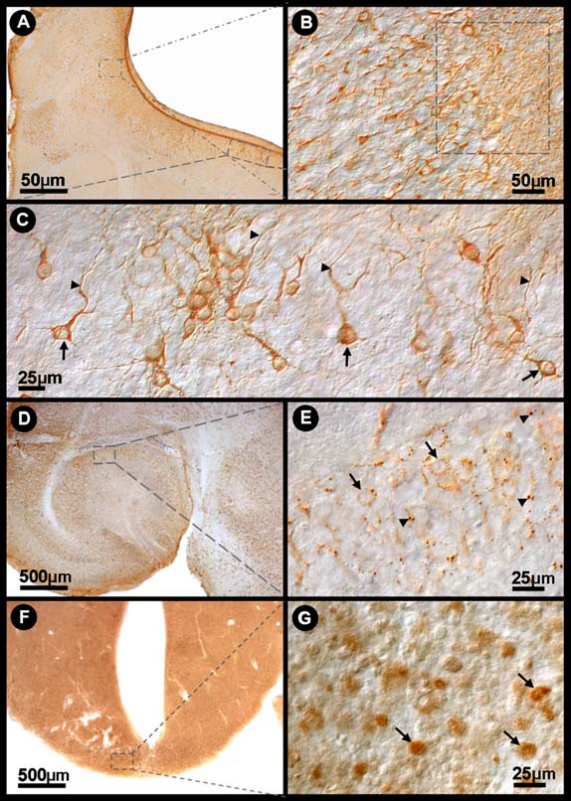
Bright-field (A, G) and interferential contrast (B–F, H) photomicrographs of infected mouse brain sections illustrating viral antigen-immunolabeled cells 2 (A–D) and 4 (E–G) days after inoculation with Curionopolis virus and TUNEL immunolabeling at 6 days (G, H). Low (square) (A), medium (B) and high (C) power photomicrographs of labeled olfactory bulb neurons. High power images illustrating isolated neurons of the olfactory bulb with immunolabeled soma (arrow) and other neuronal appendages (arrowheads) (C); immunolabeled meningeal cells are also indicated (arrow) (D); cortical (E) and thalamic (F) neurons immunostained with viral antigens distributed in the cell appendages. TUNEL-positive neurons in infected brain sections (TUNEL POD procedure) 6 days after inoculation with Curionopolis virus into the ventral olfactory bulb (G, H). The arrows indicate immunostained neuronal nuclei.

Figure 6 shows the distribution of Curionopolis viral antigens in the brain parenchyma at different time points after intranasal inoculation of the virus. The cytoplasm of infected cells stained positive for virus proteins and small unstained nuclei were apparent. This feature is consistent with the known fact that viral proteins associated with RNA viruses are located in the cytoplasm. At 1 dpi, Curionopolis antigens were detected in meningeal cells and in a few neurons located in the cortical parenchyma near the pial surface (data not shown). No clinical signs of infection were observed at this stage. At 2 dpi, clusters of immunostained neurons were clearly visible in ventral sections of the olfactory bulb, ventral prefrontal cortex and meninges. Neuronal soma, dendrites and the initial part of the axon were labeled. At 4 dpi, hypomotility and absence of milk in the stomach were frequent clinical signs, and immunostaining for virus proteins revealed a large number of infected cells. Immunolabeled primary dendrites and cell soma with altered cell appendages showing small dots of dense accumulation of viral antigens were detected in the meninges and on the pial surface of the olfactory bulb, cerebral cortex, hippocampus, inferior colliculus, thalamus and pons. Death and an agonic state were the dominant scenario in the colony at 6 dpi. Clinical signs included complete absence of milk in the stomach, weight loss, impaired body growth, meningeal irritative signs such as nuchal and thoracic rigidity, motor incoordination, spasticity and, in some cases, permanent lateral decubitus with opisthotonus after stimulation. Immunolabeling at this time point revealed viral antigens throughout the cerebral parenchyma, with intense staining of the meninges, cerebral cortex (near pial surface), CA1, dentate gyrus, inferior colliculus, pons and thalamus, and a reduced staining intensity in the cerebellum. Necrotic tissue was always detected by this histological procedure at a later stage during the disease (data not shown).

**Figure 7:**
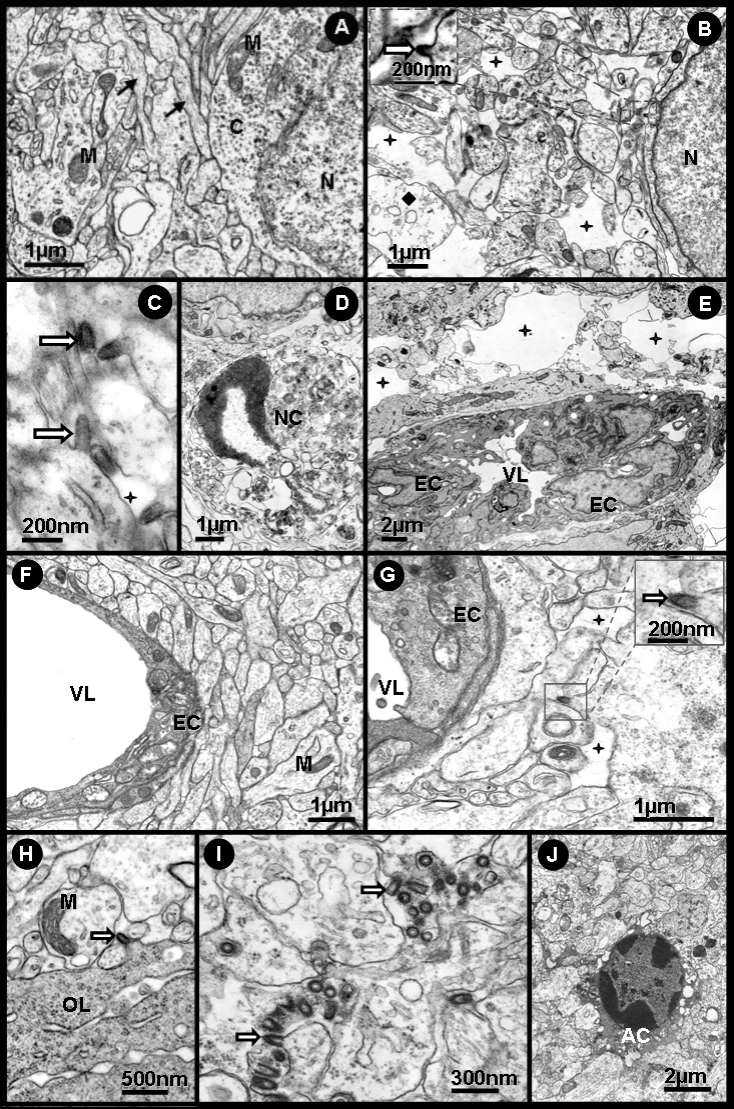
Bright-field (A, D, F) and interferential contrast (B, C, E, G) photomicrographs of Itacaiunas virus-infected mouse brain at 4 (A–C), 6 (D, E) and 8 (F, G) days post-inoculation. Low (A) and medium (B) power photomicrographs of the olfactory neuronal group (smaller rectangle). Details of immunolabeled neurons of the frontal cortex (C) (arrows and arrowheads). Low (D) (rectangle) and medium (E) power photomicrographs of a group (arrows) of hippocampal neurons showing low viral antigen condensation (arrowhead). TUNEL-positive midbrain neurons of infected brain sections (TUNEL POD procedure) 12 days after inoculation with Itacaiunas virus (F, G). The arrows indicate immunostained neuronal nuclei.

Figure 7 shows the distribution of Itacaiunas viral antigens in the brain parenchyma at different time point after intranasal inoculation of the virus. Viral antigens were only detected at 3 dpi. At this time point, a few clusters of immunolabeled neurons were found in the frontal, temporal and parietal cortices near the pial surface, but mainly in the olfactory bulb. Most labeled cells were pyramidal neurons showing frequent immunolabeling of the proximal dendrites. Axonal segments of pyramidal neurons were also visible to varying extents. The findings observed at 6 dpi were similar to those found on day 4, with the absence of clinical signs and diffuse immunolabeling, which was slightly more intense than on day 4. The full spectrum of clinical signs was observed on day 8, ranging from healthy to moribund animals, and included hypomotility, absence of suckling, weight loss, impaired body growth, nuchal rigidity, motor incoordination, and in some terminal animals, permanent lateral decubitus with opisthotonus after stimulation. Intense and diffuse CNS immunolabeling occurred, with a large number of labeled neurons in the cortical, hippocampal and pontine regions. Round immunolabeled dots were frequently detected in the cytoplasm of neuronal soma in all stained areas. These dots corresponded to the local accumulation of viral antigenic proteins.

Figure 6 (G, H) and Figure 7 (F, G) illustrate neuronal TUNEL immunolabeling on infected brain sections after inoculation of Curionopolis virus (6 dpi) and Itacaiunas virus (12 dpi). Massive TUNEL-labeled neuronal nuclei were found at this stage spread throughout many regions of the CNS, including cerebral cortices, basal ganglia, diencephalon, midbrain, cerebellum and brain stem. Astrocytes and microglial cells did not show positive TUNEL staining after infection with Itacaiunas or Curionopolis virus, indicating that these cells are activated and participate in the pathogenesis of the CNS rather than succumb to it. In contrast, oligodendrocytes seemed to be infected with Itacaiunas virus as demonstrated by transmission electron microscopy.

Table 1 summarizes the correlation between immunolabeling and clinical signs for Curionopolis and Itacaiunas virus.

**Table 1:** Clinical Signs Intensity and Virus Immunolabelling after Inoculation

Virus	Events	Days of post-inoculation survival and intensity of reported events						
		1	2	4	6	8	12	15
**Itacaiunas**	Clinical signs	(−)	(−)	(−)	(−)	(++++) (−)	(++++) (−)	(++++) (−)
	Viral antigens	(−)	(−)	(++)	(++++)	(++++)	(++++) (−)	(++++) (−)
**Curionopolis**	Clinical signs	(−)	(−)	(+)	(++++)			
	Viral antigens	(+)	(++)	(+++)	(++++)			

(−) Absence of clinical signs or viral immunolabelling; (+), (++), (+++) and (++++) distinguish progressive intensities of clinical signs and immunolabelling

A few sick animals survived up to 15 dpi and were represented by agonic individuals showing intense weight loss and health individuals whose body growth was similar to that of the control group at this time point. Healthy individuals remained as such at least until the day of sacrifice 2 weeks later and corresponded to 65% of the colony. Immunohistochemistry for viral antigens revealed massive immunostaining in agonic terminal individuals (data not shown) and no staining in healthy animals at this time point.

## Discussion

In the present study newborn mice and neuronal or glial cell cultures were infected with two species of the newly characterized genus *Bracorhabdovirus*, Itacaiunas virus and Curionopolis virus [14]. *In vivo* assays showed that after nostril inoculation infection of the meninges and olfactory bulb was followed by frontal, parietal and temporal neuroinvasion. Thereafter, virus immunolabeling spread rapidly within the brain to many other limbic areas. Cell death by apoptosis and necrosis seems to be a common neuropathological feature in both cases, but Curionopolis virus invariably produced lethal encephalitis, whereas Itacaiunas virus produced a subacute non-lethal infection in 65% of the inoculated mice. *In vitro* assays demonstrated the direct cytopathic effects of the two viruses on neurons but not on astrocytes or microglial cells. Cytopathic effects associated with viral antigens of Curionopolis or Itacaiunas virus were detected in primary neuronal cultures on day 2 post-inoculation, followed by monolayer destruction on days 5 and 7, respectively. Ultrastructural analysis revealed frequent bullet-shaped budding from the neuronal membrane for both Curionopolis and Itacaiunas viruses, a typical feature of members of the Rhabdoviridae family. Viral budding from glial cells was observed in brain tissue infected with Itacaiunas virus but not with Curionopolis virus, with the cells exhibiting a morphology similar to that of oligodendrocytes.

A variety of neurotrophic viruses can use the olfactory system as a route for neuroinvasion of the mammalian brain, e.g. herpes simplex virus 1, mouse hepatitis viruses, pseudorabies virus, Venezuelan Equine Encephalitis virus, and the challenge virus standard (CVS) strain of the rabies virus [35], [36], [1], [37], [38] but the sequence of neuroinvasion observed for Curionopolis and Itacaiunas viruses remember that of VSIV. After nasal administration, both Curionopolis and Itacaiunas antigens were observed in the meninges and olfactory bulb at 2 and 4 dpi, respectively. Six days after inoculation, strong signs of Curionopolis virus infection were found both inside, and outside the olfactory system, including the frontal cortex, hippocampus and thalamus. Itacaiunas immunolabeling was detected at 4 dpi in the olfactory bulb and in small groups of neurons of the frontal, parietal and temporal cortices near the pial surface. After Itacaiunas infection, surviving mice were immunonegative for virus antigens, suggesting a complete clearance of the virus from the brain parenchyma within 15 dpi. When VSIV is inoculated intranasally into 5–7-week-old male BALB/c mice, olfactory receptors are the first cells to be infected [10], followed by neurons of the olfactory bulb and, finally, acute infection of other brain areas [21], [8], [22]. A peak in viral antigens in the brain parenchyma, accompanied by the highest mortality rate, is observed within 7 to 10 dpi. In surviving mice, complete clearance of the virus from the parenchyma occurs within 12 dpi, without long-term damage to the brain [21], [8]. Thus, the temporal course of immunolabeling after intranasal inoculation revealing the sequence of neuroinvasion by Itacaiunas and Curionopolis viruses through the olfactory route in neonate albino Swiss mice, seem to be different of the BALB/cByJ and SJL/J neonate mice after mouse hepatitis virus (MHV)-JHM infection where MHV lesions are diffusely distributed throughout the brain suggesting blood-borne infection [39]. These data may suggest that neonate albino Swiss mice would present an effective blood-brain barrier earlier than BALB/c mice. However, since intranasal inoculation of these viruses in mature albino Swiss mice did not induce encephalitis but exhibit antigenic immunolabeling in non-symptomatic subjects (not illustrated), it remains an open question whether or not vascular route remain an alternative pathway for neuroinvasion in neonatal period. Since neurotropic viruses that enter the CNS through the vascular route may damage the blood-brain barrier it would be interesting to investigate in neonate mice, early and late in the disease, the integrity of the blood brain barrier in order to clarify this possibility. Blood brain barrier (BBB) disruption is associated with activating sentinel macrophages [40], [41], [42] and nitric oxide synthases type III in astrocytes and in endothelial or ependymal cells [43]. These macrophages may activate matrix metalloproteinases which have been associated with breakdown of the blood-brain barrier and tissue destruction [41]. Some viruses are also known to infect mouse brain endothelial cells, increasing CNS vascular permeability and resulting in edema and an increased local viral load [44], [45]. Lymphocytic infiltration and gliosis are common pathological findings in viral panencephalitis, with CD4+ T lymphocytes being observed in perivascular areas and CD8+ lymphocytes in the parenchyma. B lymphocytes are usually located in large perivascular cuffs associated with a longer and slower disease [46]. A time course study of the infection in the neonate albino Swiss mice for cellular immune response would be important to understand pathophysiological mechanisms of Itacaiunas and Curionopolis neuroinvasion.

Intense pyknosis in the cerebral cortex demonstrated by hematoxylin staining was not always accompanied by intense TUNEL immunolabeling in Curionopolis or Itacaiunas virus. Recent studies have shown an apoptosis-necrosis continuum for these two major forms of cell death [47]. However, a larger number of apoptotic cortical neurons were observed for Curionopolis virus at 72 h post-inoculation when compared to Itacaiunas virus-infected brain. This fact might be associated with the earlier cytopathic changes induced by precocious Curionopolis neuroinvasion, activating first cell death mechanisms. In addition, a previous report [11] showed that, despite intense pyknosis observed by hematoxylin staining, anti-caspase-3 labeling was consistently negative after Itacaiunas virus infection. This finding demonstrates that not all pyknotic profiles represent cells undergoing apoptosis that depends on caspase-3 activation and that apoptosis determined by TUNEL-positive DNA damage can occur irrespective of caspase-3 activation in some circumstances such as hypoxia [48]. Both necrosis and apoptosis can also be a consequence of the inflammatory response induced by the virus as previously suggested [49], [4], [50]. As reported for humans and mice, capillary and endothelial inflammation of cortical vessels is also a striking pathological feature after viral encephalitis and may contribute to blood-brain barrier damage and viral neuroinvasion [51], [52].

Ultrastructural studies have shown that the morphology of the Itacaiunas and Curionopolis viruses resembles that of animal-infecting members of the family Rhabdoviridae. Lyssaviruses have been demonstrated to frequently become enveloped virions within the endoplasmic reticulum and on the plasma membrane of infected neurons [53]. In contrast, vesiculoviruses bud from the plasma membrane of infected cells, but the process of budding into cytoplasmic vesicles was rarely observed [54], [55]. In the present study, ultrastructural analysis showed frequent bullet-shaped budding from neuronal membranes, indicating that Curionopolis and Itacaiunas viruses formed enveloped virions on the plasma membrane but not within the endoplasmic reticulum, a finding suggesting a replication process similar to that of vesiculoviruses.

Overall, our results provide at the first time fresh insights into the cell targets and the pathogenesis of two species of the newly characterized genus *Bracorhabdovirus*. We identified CNS mice cell types both in vivo and in vitro and the temporal sequence of neuroanatomical olfactory areas infected by Itacaiunas and Curionopolis virus, and our next aim is to further characterize how these viruses interact wit these cells. It will be of interest to determine the cellular associated immune response in a time course study to investigate the molecular mechanisms associated with the higher virulence of Curionopolis virus infection. It will be also useful to investigate the molecular mechanisms that made glial cells, similar to oligodendrocytes, sensitive to Itacaiunas virus, but not to Curionopolis virus infections.
